# A molecular model of the full-length human NOD-like receptor family CARD domain containing 5 (NLRC5) protein

**DOI:** 10.1186/1471-2105-14-275

**Published:** 2013-09-17

**Authors:** János András Mótyán, Péter Bagossi, Szilvia Benkő, József Tőzsér

**Affiliations:** 1 Department of Biochemistry and Molecular Biology, Faculty of Medicine, Medical and Health Science Center, University of Debrecen, POB 6, H-4012 Debrecen, Hungary; 2 Department of Physiology, Faculty of Medicine, Medical and Health Science Center, University of Debrecen, Debrecen, Hungary

**Keywords:** NLRC5, Molecular modeling, LRR protein, NOD-like receptor

## Abstract

**Background:**

Pattern recognition receptors of the immune system have key roles in the regulation of pathways after the recognition of microbial- and danger-associated molecular patterns in vertebrates. Members of NOD-like receptor (NLR) family typically function intracellularly. The NOD-like receptor family CARD domain containing 5 (NLRC5) is the largest member of this family that also contains the largest number of leucine-rich repeats (LRRs).

Due to the lack of crystal structures of full-length NLRs, projects have been initiated with the aim to model certain or all members of the family, but systematic studies did not model the full-length NLRC5 due to its unique domain architecture.

Our aim was to analyze the LRR sequences of NLRC5 and some NLRC5-related proteins and to build a model for the full-length human NLRC5 by homology modeling.

**Results:**

LRR sequences of NLRC5 were aligned and were compared with the consensus pattern of ribonuclease inhibitor protein (RI)-like LRR subfamily. Two types of alternating consensus patterns previously identified for RI repeats were also found in NLRC5. A homology model for full-length human NLRC5 was prepared and, besides the closed conformation of monomeric NLRC5, a heptameric platform was also modeled for the opened conformational NLRC5 monomers.

**Conclusions:**

Identification of consensus patterns of leucine-rich repeat sequences helped to identify LRRs in NLRC5 and to predict their number and position within the protein. In spite of the lack of fully adequate template structures, the presence of an untypical CARD domain and unusually high number of LRRs in NLRC5, we were able to construct a homology model for both the monomeric and homo-heptameric full-length human NLRC5 protein.

## Background

The innate immune system in vertebrates has pattern recognition receptor (PRR) families that trigger inflammatory pathways in response to microbes or danger signals. Both the secreted PRRs released into the extracellular space as well as the membrane-associated PRRs, like Toll-like receptors (TLRs), are responsible for the recognition of extracellular pathogen associated molecular patterns (PAMPs), while the PRRs located in the cytosol, like NOD-like receptors (NLRs), recognize PAMPs intracellularly [1].

**Figure 1:**
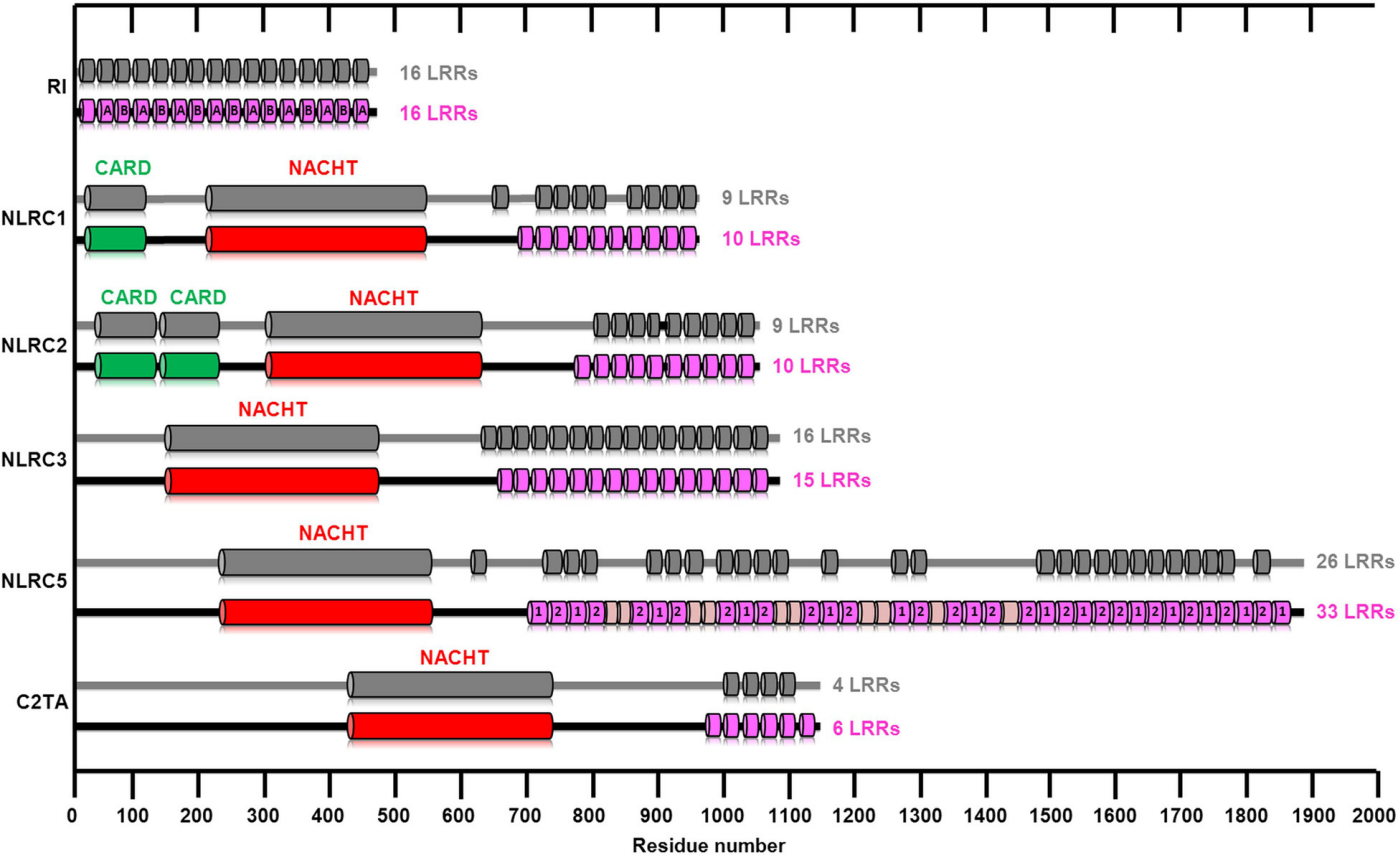
**Schematic representation of the domain structures of NLRC5 and NLRC5-related proteins.** Upper lines in grey color: Domain architecture of RI [PDB: 1Z7X]; NLRC1 [UniProtKB: Q9Y239]; NLRC2 [UniProtKB: Q9HC29]; NLRC3 [UniProtKB: Q7RTR2]; NLRC5 [UniProtKB: Q86WI3] and C2TA [UniProtKB: P33076] proteins based on data available in the PDB and the UniProtKB databases at 22th January 2013. Under lines: Domain architecture of NLRC5 and NLRC5-related proteins; the CARD and NACHT domains are indicated based on the database data and the putative LRRs are indicated based on the results of the sequence alignment and consensus sequences. uCARD domain of NLRC5 is not shown, because this domain is not indicated in the UniProt database due to its untypical nature. Domain and color legend: CARD - green, NACHT - red, LRR – magenta. Typical consensus sequences of LRRs of RI (type A and B) and NLRC5 (type 1 and 2) of NLRC5 proteins and total number of LRRs are indicated. The LRRs of NLRC5 which did not fit to the consensus pattern and length of RI-like LRR sequences are indicated with brighter magenta color.

In humans, the NLR family contains 22 members having in common the presence of a central NACHT domain (present in NAIP, CIITA, HET-E, and TP1) and a C-terminal leucine-rich repeat (LRR) receptor domain with various lengths [2-5] (Figure 1). Classification of NLR protein family members is based on the type of the N-terminal effector domains that defines the subfamilies of the NLR family. For example, the NLRC proteins contain CARD domain (caspase activation and recruitment domain), the NLRP proteins contain PYRIN domain, while the NAIP protein (neuronal apoptosis inhibitory protein, also referred as NLRB1) has BIR (baculovirus inhibitor of apoptosis protein repeat) domain [4]. The NLRC subfamily contains five proteins. NLRC1 (nucleotide-binding oligomerization domain containing protein 1, also referred as NOD1) and NLRC2 (NOD2) possess one and two CARD domains, respectively [4]. Though the CARD domain of NLRC3 (NOD4), C2TA (MHC class II transactivator protein, also referred as CIITA or NLRA) and NLRC5 (NOD-like receptor family CARD domain containing 5) is well conserved in the mammalian orthologs [6], they show low sequence similarity to the „typical” CARD domains. Therefore they are classified into an untypical CARD domain-containing subfamily of NLRs [4,7]. The untypical CARD domain of NLRC5 is referred in this paper as uCARD domain.

Based on the alignments of N- and C-terminal domain sequences [6,8] the NLRC1, NLRC2, NLRC3 and C2TA proteins of NLR protein family are the most closely related to NLRC5. Alignment of the N-terminal effector domains, the NACHT and LRR domains of NLRs showed that C2TA protein shows the highest similarity to NLRC5 among the NLRC5-related proteins [4,6,8].

NLRC5 is the largest family member of NLR family, containing 1866 amino acid residues that results in 204 kDa predicted molecular weight [6]. Recent intensive studies of this receptor revealed controversial roles for this protein in both the innate and adaptive immune responses.

In the domain architecture of NLRC5 the uCARD domain is followed by NACHT, winged helix (WH), superhelical (SH) and LRR domains [4]. The NACHT domain is also known as nucleotide-binding domain (NBD) or nucleotide-binding oligomerization domain (NOD) and has an important role in the oligomerization of NLRs, while the signaling effector uCARD domain is predicted to be responsible for the interactions with the adaptor proteins and for the activation of downstream signaling [7]. Furthermore, it also has a key role in the nuclear import since it contains the nuclear localization signal (Lys121-Arg122-Arg132-Arg133-Lys134 in NLRC5 numbering) [5]. Most NLR protein family members contain highly conserved residues in their WH domain, while the function of SH domain (containing eight α-helices) is currently unknown [4]. The C-terminal end of the NLRC5 contains LRRs that are typically 20–30 residue-long, rich in leucine and contain consensus sequence motifs [9,10]. The hydrophobic residues are located in the inner part of the LRR structural motif, while the hydrophilic residues are exposed to the outer side of the repeats [10]. Importantly, LRR-containing proteins differ in the number of consecutive LRRs. LRR structures generally adopt a “horseshoe” shape, in which the β-sheets of LRRs are on the concave side and the helical elements are on the convex side of the curved shape [9,10]. LRR domains are thought to be responsible for the interactions with ligands; however, the structural basis of specific molecule recognition is not known [4]. The ribonuclease inhibitor (RI) protein of *Sus scrofa* was the first LRR-containing protein of which crystal structure was solved, providing an insight into the structure of LRRs [11]. Despite the large number of currently available crystal structures of LRR-containing proteins [10], there is no available structural data for full-length NLRs. While NMR or X-ray crystallographic structures of CARD or PYRIN domains of NLRC or NLRP subfamily members are available, only short regions of LRR sections of NLRs have been solved up to now. The lack of structural data of NLRC5 initiated both individual [12] and systematic studies [4,8] to generate homology models. A previous systematic study hadn’t prepared a homology model for NLRC5, noting that its LRR domain appeared to be extremely large compared to other LRR-containing proteins and the potential template structures were found to be too small for homology modeling [8]. Therefore, the goal of our study presented here was to predict the structure of both the monomeric and the heptameric form of the full-length human NLRC5 protein.

## Methods

### Databases and sequence alignments

Structural data were downloaded from Protein Data Bank [13] while protein sequences and sequence annotation data were derived from UniProt database [14]. Multiple sequence alignments were performed using Clustal W (1.83) and Clustal X (2.0.12) programs [15]. The LRRML conformational LRR database (v0.6) [16], the LRRfinder2.0 webserver [17] and the LRR Conservation Mapping Program [18] were used for the analyses of LRRs of NLRC5 protein.

### Molecular modeling

Secondary structure predictions were performed using the PredictProtein server [19]. Modeller 7v7 software was used to prepare the homology models [20]. Molecular dynamics calculations were performed by Sybyl program package (Tripos Inc., St. Louis, MO, USA) using the following parameters: AMBER7_FF99 force field, 1 fs step size, 6 Å non-bonded cutoff, dielectric constant was set to 4 and only the SH-LRR linker region of human NLRC5 (Thr653-Gln687) was allowed to move. The molecule was gradually cooled from 300 to 10 K temperature (temperature was set to 300, 250, 200, 150, 100 and 10 K in the consecutive steps) and 1000 fs-long runs were performed on every temperature value. Energy minimizations were performed by Sybyl without any fixed atoms using the following parameters: AMBER7_FF99 force field, 100–100 Powell iterations were applied using 6, 7 and 8 Å non-bonded cutoff values in the consecutive steps and the dielectric constant was set to 4, 3 and 1, respectively, followed by further 10.000 iterations using the parameters of the last previous run. Calculations and molecular visualizations of the structural models were performed using the Sybyl program package run on Silicon Graphics Fuel workstations (Silicon Graphics International, Fremont, CA, USA). POLYVIEW-3D (available at: http://polyview.cchmc.org/polyview3d.html) was used to prepare surface representation [21].

## Results and discussion

### Sequence alignment of NLRC5 and NLRC5-related proteins

Previously published phylogenetic analyses were used to identify the most closely related NLR proteins from which the C2TA showed the highest sequence similarity to NLRC5 [6,8]. Using structural and sequence data available in databases, first we compared the domain organization of the RI**,** C2TA, NLRC1, NLRC2, NLRC3 and NLRC5 proteins (Figure 1). Our result confirmed that NLRC5 shows a tripartite domain structure and was predicted to consist of an N-terminal uCARD domain followed by the NACHT domain, and the C-terminal part of the protein contains LRRs [22]. The domain organization of NLRC1, NLRC2, NLRC3 and NLRC5 proteins is very similar, but while NLRC1 contains one and NLRC2 contains two typical CARD domains, the NLRC3 and NLRC5 proteins contain an untypical CARD domain [4].

**Figure 2:**
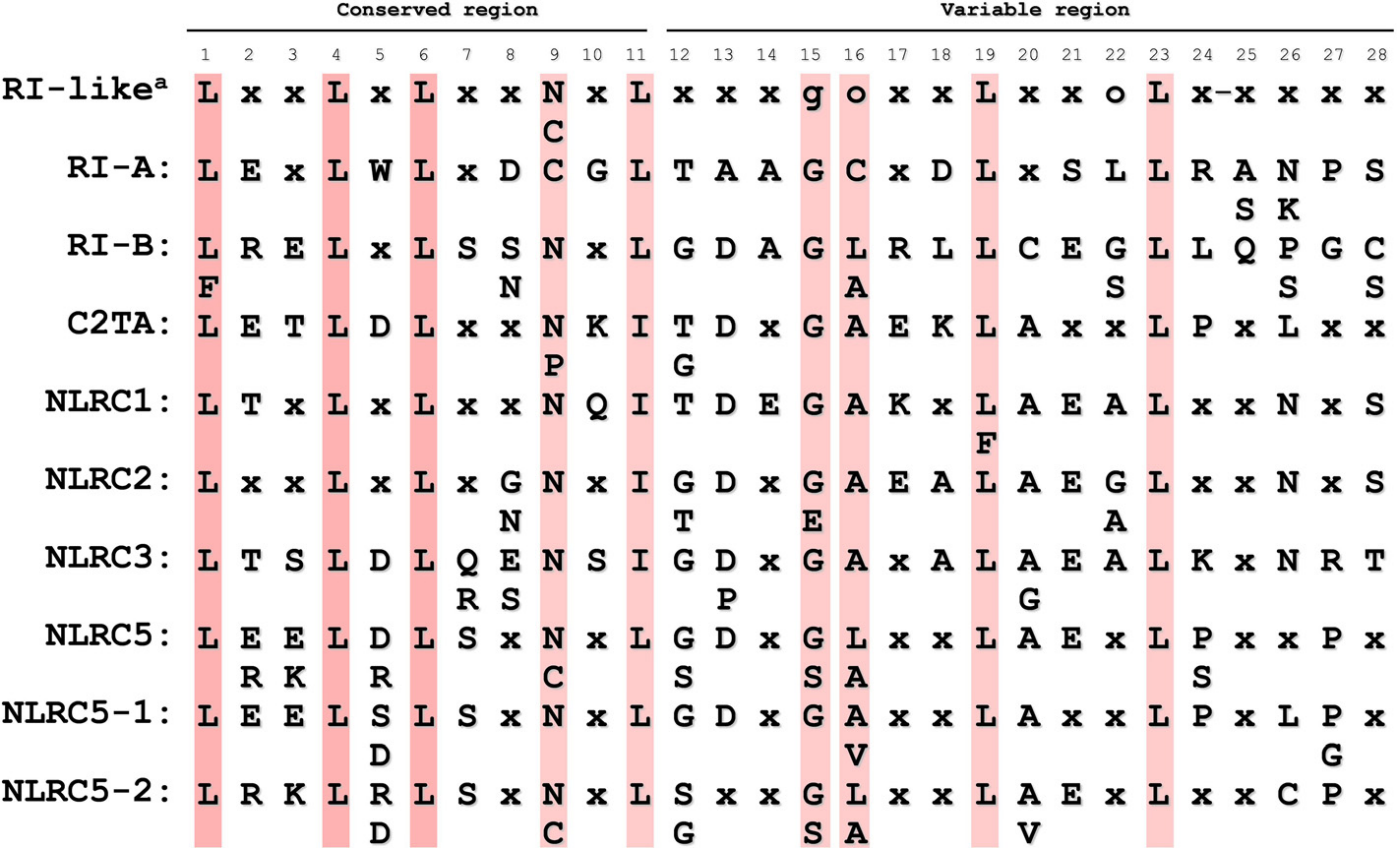
**The consensus sequences of putative C-terminal LRRs of NLRC5 and NLRC5-related proteins.** Conserved residues are marked by pink background and „X” represents any residue. Synonymous amino acids: L, I and V; S and T; E, D, Q and N; R and K. Most frequent amino acids are shown within or under the consensus lines. Two typical types of LRRs are represented by A and B in case of RI and by 1 and 2 in case of NLRC5. ^a^Consensus pattern of LRR proteins belonging to RI-like subfamily based on Bella et al. [10]. „o” represents a nonpolar residue, „-” represents a possible insertion site, g represents Gly residue identical or conservatively substituted in its positions in more than 30% of sequences.

To find consensus patterns in the sequences of leucine-rich repeats we analyzed the amino acid sequences of RI, C2TA, NLRC1, NLRC2, NLRC3 and NLRC5 proteins. Sequence alignment was performed using the sequences of putative LRRs of NLRC5 and NLRC5-related proteins of different mammalian origin (human, mouse, horse, bovine, pig, dog, rat or rhesus macaque) (Figure 2).

Leucine-rich repeats of LRR-containing proteins belonging to RI-like subfamily are typically 28–29 residues long, having α-helical conformation in their convex side and show a high degree of curvature [9,10]. Our result from sequence alignment and LRRML Conformational LRR XML-Database analysis [16] showed that the LRRs of NLRC5 and RI, C2TA, NLRC1, NLRC2 and NLRC3 proteins are predominantly large (28–29 amino acid long). Furthermore, we found that the consensus sequences of LRRs correspond well to the consensus pattern of RI-like LRR subfamily (Figure 2) classified by Kajava and Kobe [9,23]. To further identify the LRR motifs, a secondary structure prediction of the full-length human NLRC5 protein was also performed using the PredictProtein server (see Additional file 1). Based on these results we determined the consensus sequences of LRRs (Figure 2) and 33 LRRs of NLRC5 were found to fit the consensus pattern and length of RI-like LRR sequences (Figure 1). Both the number of LRRs and their boundaries obtained in this way differed from the data available in the databases (Figure 1).

Human NLRC5 gene contains 49 exons from which 43 (from exon 7 to exon 49), being part of the transcript of the coding sequence [Ensembl: ENST00000262510], were analyzed. We observed that exons start at position 6 of the previously showed consensus sequence of LRRs. Out of the 43 exons analyzed, seven (exons 11, 12, 16, 17, 21, 26 and 30) were found to be too short and/or did not fit the consensus pattern. These findings were confirmed by the analysis using the LRR Conservation Mapping Program [18], therefore the exon structure analysis predicted the presence of 36 typical LRRs in NLRC5.

In the absence of crystal structures the number and individual length of LRRs in NLRC5 can only be predicted with sequence annotation methods that are imprecise [10]. Therefore, NLRC5 was predicted previously to contain 20 LRRs [22], more than 20 LRRs [24], 26 LRRs (http://www.uniprot.org), 27 LRRs [6] or 43 LRRs [8] in different studies.

To gain more insight to LRRs we have also utilized other bioinformatical tools: the LRRfinder2.0 webserver for the prediction of leucine-rich repeats [17], the LRRML Conformational LRR XML-Database [16] and the LRR Conservation Mapping Program [18]. LRRfinder2.0 identified the lowest number of LRRs in NLRC5 which showed the typical length of RI-like leucine-rich repeats. On the other hand, the LRRML Conformational LRR XML-Database was apparently more suitable to identify consensus patterns in the LRRs, and the results we obtained by the recently developed LRR Conservation Mapping Program were in agreement with the results of exon analysis, that we described above.

In spite of the use of multiple approaches in our study, the exact number of LRRs in NLRC5 is still uncertain and is likely between 33 and 43.

To predict the presence of unstructured regions, the sequence of NLRC5 [UniProtKB: Q86WI3] was analyzed by using the IUPred web server [25]. We found that Ala822-Ser842 and Gly953-Ser962 regions of the LRR domain could be short disordered regions. Interestingly, these regions are encoded by the parts of exons 11, 12, 16 and 17, which were excluded as LRRs from the sequence alignment due to their short length and lack of consensus pattern. Although, alignment by the ANCHOR web server [26] did not predict disordered binding regions for the same regions of the LRR domain, we hypothesize that these short disordered regions could be responsible for the interactions with the ligands of NLRC5. However, experimentally determined structural data are required to verify the proposed disordered nature of these regions, as well as the true number, length and boundaries of LRRs.

With further analysis of the LRR consensus patterns we identified two types of consensus sequences (type 1 and 2) in NLRC5 (Figure 2). It was shown that these consensus sequences alternate in the consecutive LRRs as it was found previously for type A and B consensus sequences of porcine ribonuclease inhibitor protein [27] (Figure 1).

Several extracellular LRR proteins were reported to contain N- and C-terminal capping motifs where disulphide-bonds stabilize and protect the structure [10]. Our sequence alignment resulted in the identification of four cysteine-rich regions within the LRR domain. The Cys648-Cys698 cysteine-rich region showing the C*x*_*23*_C*x*_*5*_C*x*_*6*_C*x*_*12*_C pattern is located at the N-terminal end of the LRR domain and possibly corresponds to an N-terminal capping motif of the LRR domain of NLRC5. The C-terminal LRR repeat of NLRC5 was found to be longer than the internal repeats (36 amino acid residue long) based on the exon analysis, and similarly to the 34 amino acid residue long C-terminal capping motif of RI protein [27] it possibly corresponds to the C-terminal capping repeat in NLRC5.

While the TLR capping motifs are well studied [28], there is no detailed information about the NLR capping motifs due to the restricted number of structural data for NLRs. Therefore, the cysteine-rich regions of human NLRC5 were not further investigated in this work. Deeper structural insight into the LRRs of NLR family members is needed to verify both the structure and the function of these cysteine-rich regions in NLRC5.

### Modeling of the full-length human NLRC5 protein

#### *Modeling of the LRR domain of human NLRC5 protein*

**Figure 3:**
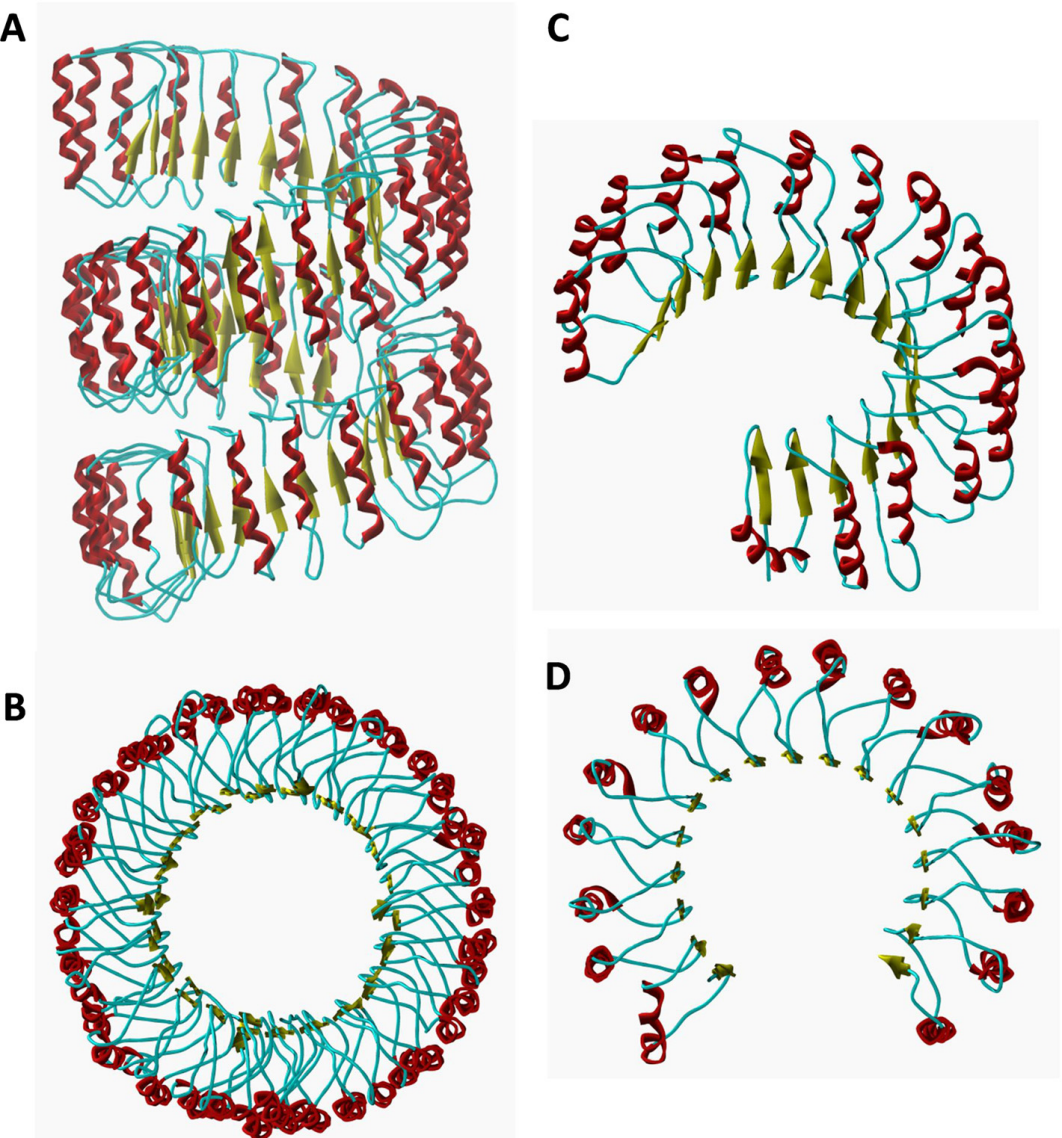
**LRRs of human NLRC5 and RI proteins.** LRR domains are shown by ribbon/tube representation both in case of the homology model of human NLRC5 (Ile688-Thr1866) **(A**,**B)** and the crystal structure of human RI (Ser1-Ser460) [PDB: 1Z7X] **(C**,**D)**. Side (upper part) and top views (lower part) are represented. Color codes: α-helix: red, β-sheet: yellow, loop: cyan.

Based on the X-ray structure of human ribonuclease inhibitor [PDB: 1Z7X] [29] we have built a model for the LRR domain of human NLRC5 (Ile688-Thr1866) by homology modeling (Figure 3A and 3B) using Modeller 7v7 software (see Additional file 2 and Additional file 3).

We have chosen to build up 43 LRRs, the largest number so far predicted, in correspondence with the study of Istomin and coworkers [8] and in agreement with the results of PredictProtein analysis (see Additional file 1). Secondary structure prediction assigned α-helices or β-sheets with slightly lower probability for the LRRs encoded by the shorter exons (exons 11, 12, 16, 17, 21, 26 and 30 exons). However, our prediction did not suggest extended unstructured regions within the LRR domain or between the consecutive LRRs. In our model the shorter LRRs were predicted to contain shorter α-helices in their convex side compared to the longer continuous α-helices of other LRRs or were predicted to contain shorter loops. Our model suggests that the shorter LRRs do not interrupt the curved shape of the LRR domain of NLRC5, similarly to the model of Neerincx and his coworkers [12] where the helical conformation of LRR domain is also continuous.

The LRR domain was suggested to adopt a large helical conformation in which the leucine-rich repeats form two full circles (Figure 3A and 3B) and does not have the previously proposed planar circular shape with large radius [8]. Our model suggests that the LRR domain of human NLRC5 does not consist of more connected circular parts and adopts a continuous LRR helix shape formed by two full LRR circles (Figure 3A and 3B) instead of the presence of only one LRR circle [12].

Both N- and C-terminal cap regions are well-studied in the case of LRR-containing proteins [10] and in TLRs [28], while there is only limited information about the capping motifs of NLRs due to the lack of crystal structures and extended sequence alignments on capping motifs of NLR family members.

The domain positioning of human NLRC5 protein was predicted based on the structure of Apaf-1 protein (Apoptotic Protease-Activating factor 1) which is distantly related to NOD-like receptors [4]. Apaf-1 was found previously to be a useful template to model the structure of NLRC5 [12], as it is a homolog of NLRC5. Structure of Apaf-1 have already been solved and was found to share the common domain organization with NLR protein family members [4]. Both a closed and an opened conformational structure of NLRC5 were predicted, therefore, two different template structures were used to predict the structure of the N-terminal domains of NLRC5 protein by homology modeling (see Additional file 2 and Additional file 3).

#### *Modeling of the N-terminal domains of the closed conformational human NLRC5 protein*

**Figure 4:**
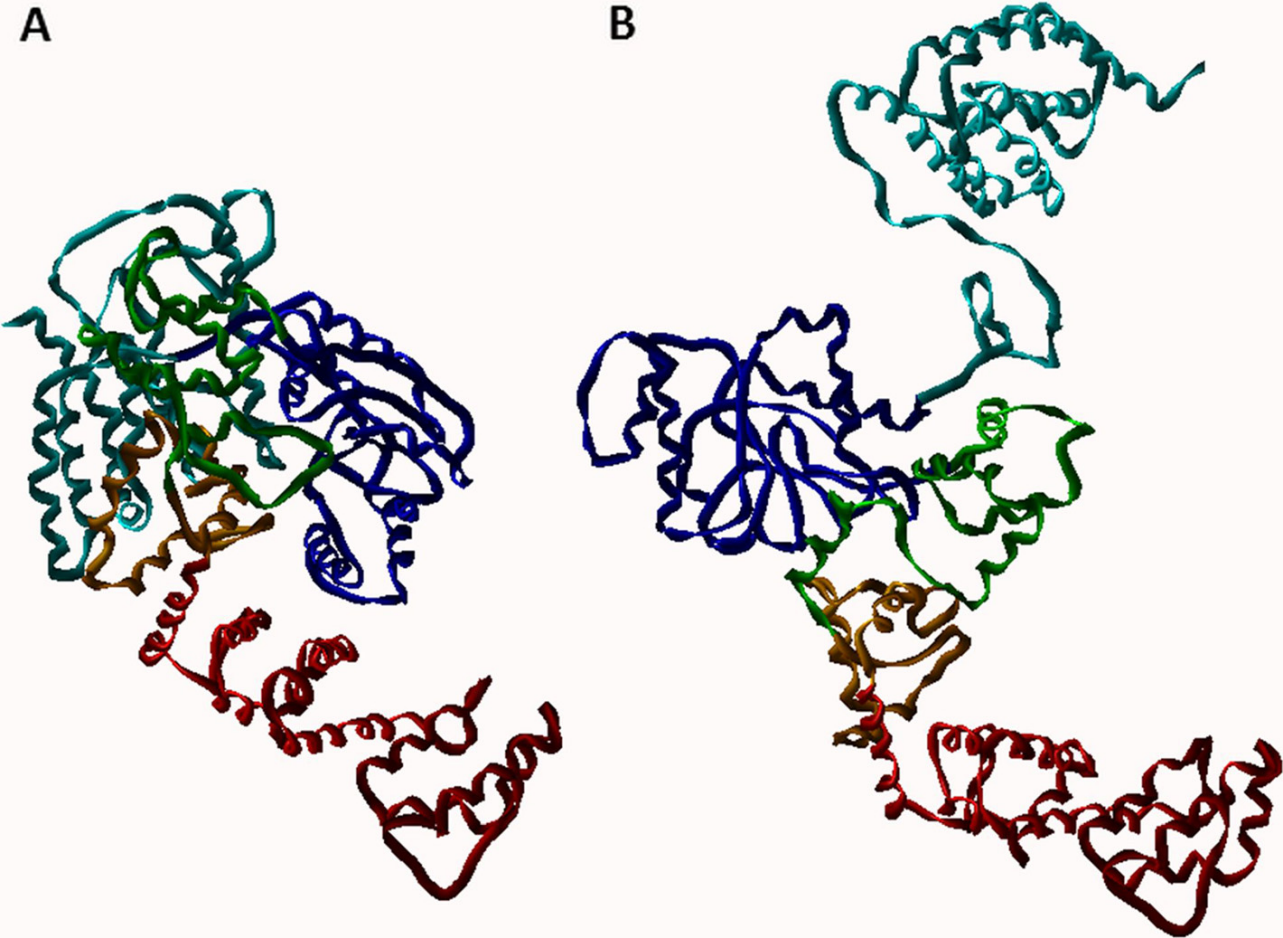
**N-terminal domains of monomeric human NLRC5 protein.** Predicted conformation of closed **(A)** and opened **(B)** conformational states are shown by ribbon representation. Domain and color codes in the homology model of human NLRC5 (Met1-Ala687): uCARD: cyan, NACHT: blue, AxP: green, WH: orange, SH: red.

Based on the crystal structure of Apaf-1 protein we have predicted a closed conformation of monomeric human NLRC5 (Met1-Ala652) (Figure 4A) using Modeller 7v7 software (see Additional file 2 and Additional file 3). The completion of the 1st, 3rd, 6th and 7th α-helices of Apaf-1 protein [PDB: 1Z6T] [30] was needed before homology modeling. Biopolymer module of Sybyl was used to complete these α-helices of the Apaf-1 which are part of the uCARD domain of human NLRC5.

The small helical domain (Pro370-Pro453) of human NLRC5 (referred here as AxP domain) is located next to the NACHT domain and contains the conserved Ala404-Val405-Pro406 structural motif. The AxP domain of NLRC5 corresponds to the small helical domain (HD1) of Apaf-1 [30] and the AxP signature corresponds to the conserved GxP structural motif of Apaf-1. Most members of NLR family contain this structural motif where only the proline residue interacting with bound ATP is highly conserved [4]. The functionally important regions of Apaf-1 superimpose well with the same regions in the predicted structure of human NLRC5: Walker A motif responsible for nucleotide triphosphate binding (Gly228-Thr235 using NLRC5 numbering) (GKAGMGKT), Walker B motif responsible for nucleotide triphosphate hydrolysis (Leu303-Leu313) (LLIFDGLDEAL), Sensor 1 (Thr345-Arg347) (TSR), WH conserved histidine (His491) and WH consensus sequence (Phe475-Ile480) (FYAKDI) based on Proell et al. [4].

#### *Modeling of the opened conformation of human NLRC5 protein*

Apoptosome is a molecular platform which mediates the proteolytic processing of procaspases during apoptosis. The apoptosome assembly is prompted by the activation of Apaf-1 protein by its conformational changes and oligomerization [31]. As Apaf-1 heptamerizes in the apoptosome complex, an opened conformation of heptameric human NLRC5 was predicted by homology modeling using the structure of the apoptosome-procaspase-9 CARD complex [PDB: 3IYT] [31] as template (see Additional file 2 and Additional file 3).

CARD domain of Apaf-1 seemed to be disordered in the crystal structure of apoptosome-procaspase-9 CARD complex [31]. Therefore, the uCARD domain of opened conformational NLRC5 (Met1-Gly197) was built up using the predicted structure of uCARD domain of closed conformational NLRC5.

The oligomerization is mediated by the NACHT domain of NLRC5 (Asp198-Gly369) as in case of Apaf-1 [31]. Following construction of the heptameric NLRC5 protein a superimposition was observed between the NACHT domains of the monomers and the uCARD domains of the neighboring monomers, while overlap of NACHT-NACHT or uCARD-uCARD domains of neighboring monomers were not present. Therefore, the Val175.n-Gln174.c-Gln174.ca-Gln174.n torsion angle was set from 155.1° to 90° within the uCARD-NACHT linker region with which an opened conformation of monomeric NLRC5 was generated (Figure 4B). We predicted that this movement of uCARD domain (Figure 4A and 4B) is needed for the activation and allows the oligomerization of NLRC5. Similar rearrangement of CARD domain was seen in case of Apaf-1 protein during the activation and formation of the heptamer [30].

**Figure 5:**
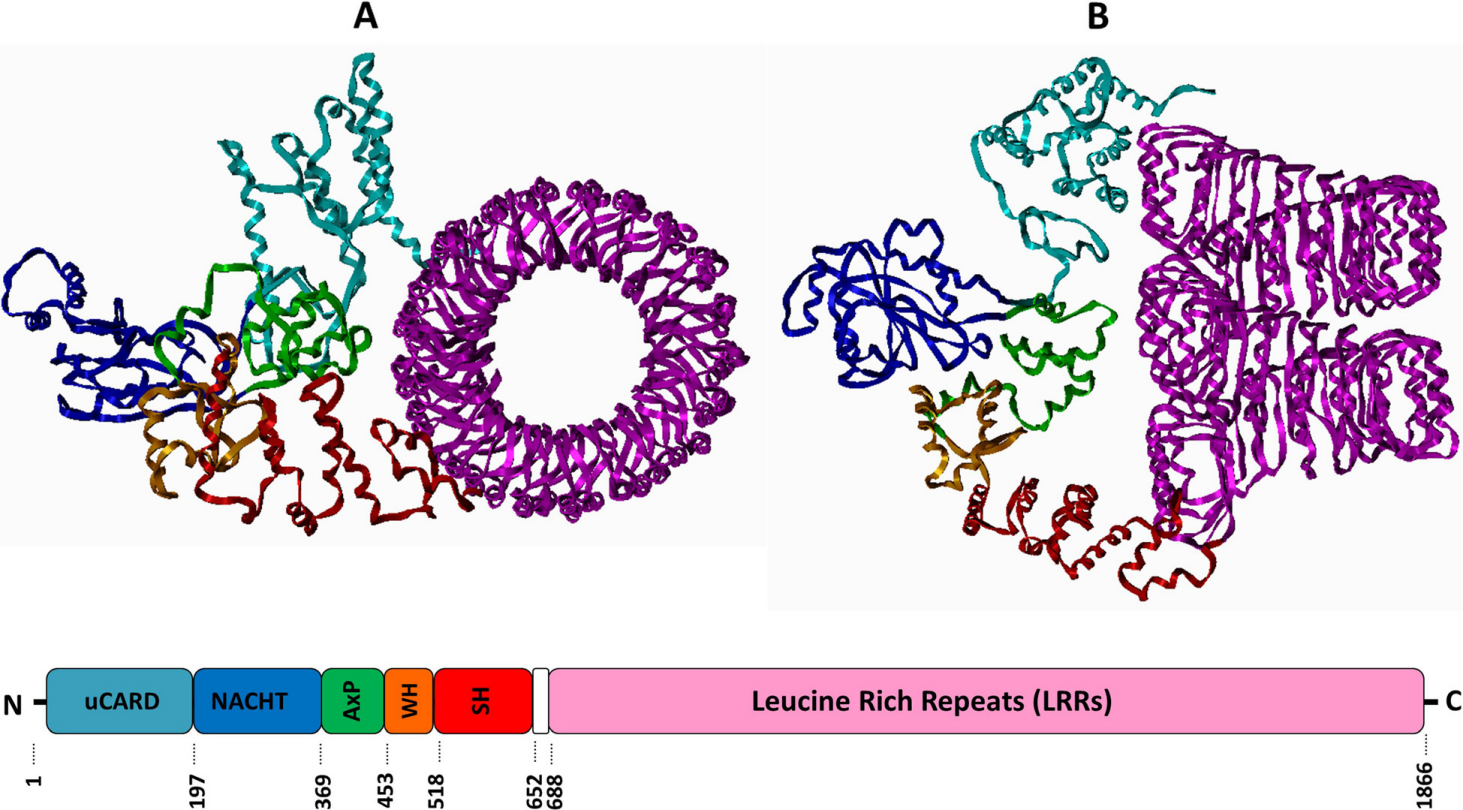
**Molecular model for the monomeric full-length human NLRC5 protein.** Top view **(A)** and side view **(B)** of the model of monomeric full-length human NLRC5 protein in its opened conformation by ribbon representation. Schematic representation of human NLRC5 structure is shown in the lower part of the figure. Domain and color codes: uCARD: cyan, NACHT: blue, AxP: green, WH: orange, SH: red, LRR: magenta.

**Figure 6:**
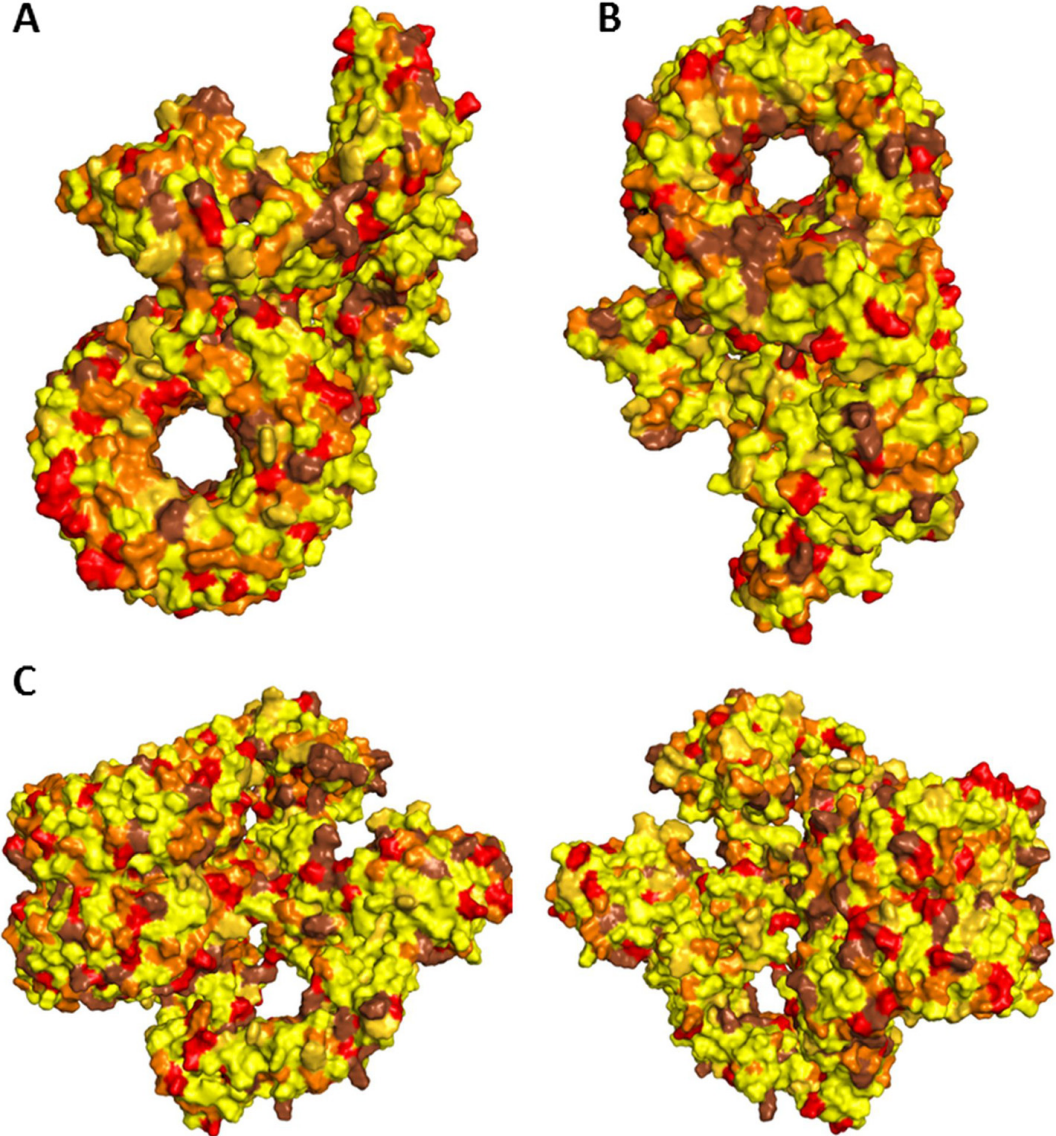
**Surface representation of monomeric full-length NLRC5.** Top **(A)**, bottom **(B)** and side views **(C)** of modeled monomeric full-length NLRC5. Color codes: hydrophobic (yellow), amphipathic (dark yellow), polar (orange), negatively charged (red), positively charged (brown).

Structure of the LRR domain of human NLRC5 was predicted in this work based on the X-ray structure of human RI protein as described above. This LRR domain (Ile688-Thr1866) was joined to the opened conformational NLRC5 (Met1-Ala652) by a linker region (Thr653-Gln687) located between the SH and LRR domains. Structure of the SH domain was completed manually by using Sybyl with the duplication of a four α-helix containing region (Gln518-Lys606) of SH domain. This duplicated part was merged into the structure as the continuation of the α-helices of the SH domain (from Leu599) and also served as a template to predict the structure of the lacking SH-LRR linker region. The predicted structure of this short region has a lower certainty, as it is expected to have very high flexibility to facilitate the rearrangement of the domains during the activation. The C-terminal end of the Thr653-Gln687 region was close to the N-terminal end of the LRR domain (Ile688-Thr1866) in its predicted spatial position, therefore these terminal amino acids were connected. A possible conformation of the SH-LRR linker region (Thr653-Gln687) was optimized by a molecular dynamics procedure. Molecular dynamics run on this linker region was followed by short energy minimization of the entire molecule without any fixed atoms to remove the unfavorable interactions. The generated structure of the monomeric full-length human NLRC5 is shown in Figure 5. The structure was refined by a longer energy minimization of the entire molecule and Sybyl program was used to identify those atoms that potentially participate in hydrogen bond formation. Based on our model the structure of monomeric NLRC5 is stabilized by LRR-GxP and LRR-CARD interdomain interactions. We found that in case of the investigated hydrogen bonds the donor-acceptor distances became close to 3.0 Å during the minimization like those being between the LRR circles. These interacting residues of LRR domain are located in the loop regions of the LRR motifs and can be found mainly in the concave sides of the curved shape, near to the inner side of the LRR helix. The hydrophobic residues of the LRRs can be found mainly in the core of these structural motifs and the charged residues are mainly exposed to the solvent (Figure 6).

It was described that the amino acid residues (Lys121-Arg122-Arg132-Arg133-Lys134) forming the nuclear localization signal (NLS) of NLRC5 are located in the uCARD domain [5]. In our model the NLS is exposed to the solvent both in the opened and closed conformation, but it is presumably less accessible in the opened conformation due to its proximity to the C-terminal end of the LRR domain. Based on the predicted structure, the NLS is not buried in the case of the closed conformational state; therefore, the domain rearrangement during the activation does not appear to be necessary for the accessibility of NLS.

**Figure 7:**
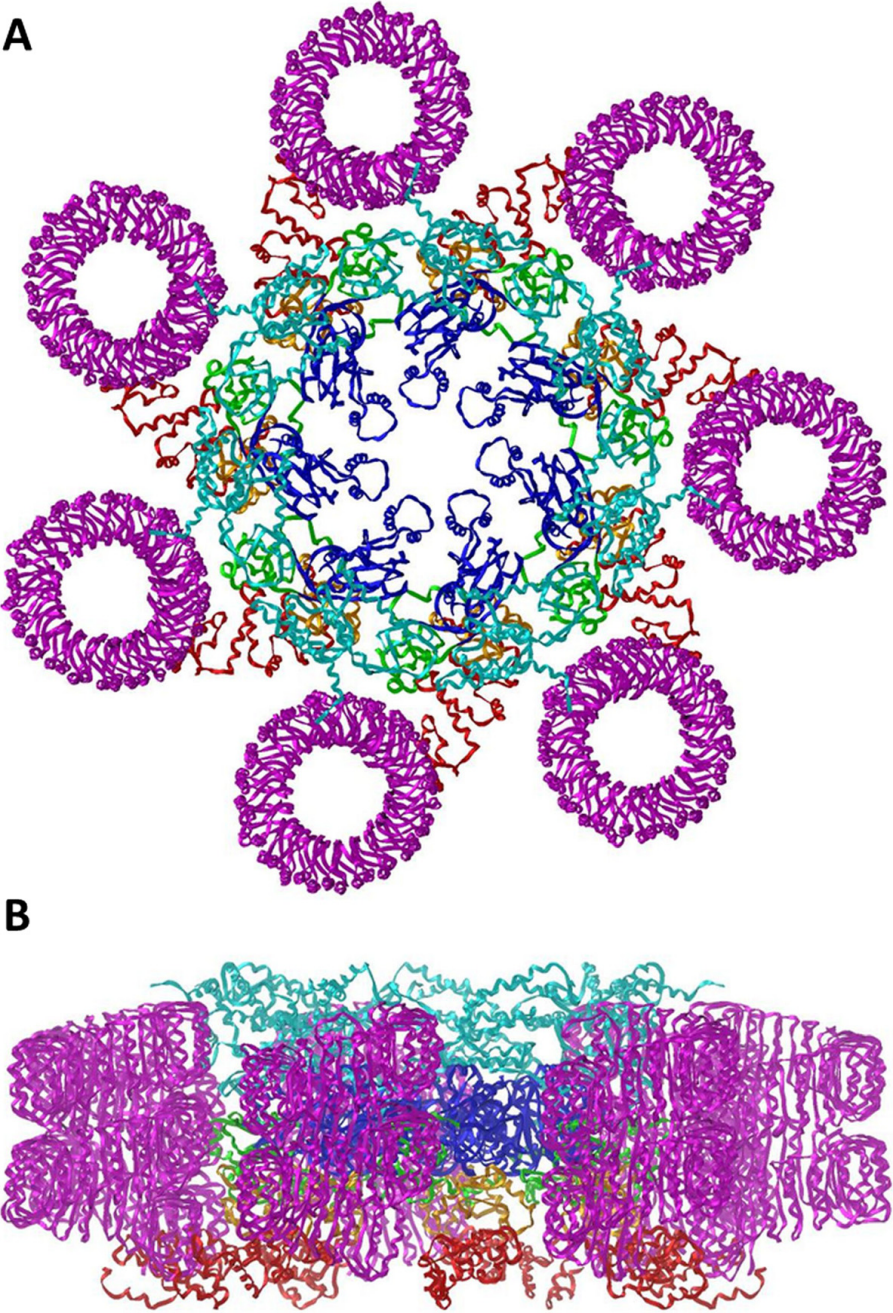
**A model for heptameric full-length human NLRC5.** Top **(A)** and side views **(B)** are represented. Domain and color codes: uCARD: cyan, NACHT: blue, AxP: green, WH: orange, SH: red, LRR: magenta.

Structure of heptameric full-length human NLRC5 protein (Figure 7) was built up from the opened conformational NLRC5 monomers using the heptameric Apaf-1 protein as template structure. Domain architecture of NLRC5 is similar to Apaf-1, which made the Apaf-1 a useful template for homology modeling. It was described that the oligomerization of these proteins depends on the hydrolysis of a nucleotide triphosphate (e.g. ATP) which leads to conformational changes, and the subsequent rearrangement of some domains is needed for the formation of the oligomeric platform [4]. It was revealed previously that most members of NOD-like receptor family share the main structural and oligomerization properties with Apaf-1 [4], therefore, we propose that activation leads to the formation of a homo-heptamer of NLRC5.

In the model of full-length human NLRC5 the axes of the heptameric platform and the LRR helices appear to be parallel and the thickness of heptameric disc and the height of LRR domains is very similar (Figure 7). In the homo-heptameric NLRC5 there are large interacting surfaces between the LRR and uCARD domains and between the AxP and SH domains. The uCARD-NACHT intramonomeric interactions of the closed conformational monomeric protein can be broken during the activation (as in case of Apaf-1 [31]), which can be compensated by the development of NACHT-NACHT and uCARD-LRR interactions during the assembly of the heptameric platform.

In this work both the closed and opened conformational states of full-length human NLRC5 protein were modeled by homology modeling. Only short energy minimization was performed on the opened conformational NLRC5 protein to study the intradomain interactions within the full circles of LRR domain, and some interdomain interactions within the monomeric NLRC5 protein. Although, the study of the domain rearrangement of NLRC5 during its activation was out of the scope of this study, we plan to perform extended molecular dynamical calculations in the future to study the motions of the NLRC5. Those molecular dynamical calculations could help us evaluate the development of the intermonomeric interactions and the oligomer formation.

Our model presented here is in good agreement with the previously proposed activation mechanism of NLR proteins [32]. The NLRC5 undergoes conformational changes during activation and an opened conformational state is established from the closed conformational (Figure 4A and 4B), allowing the formation of a homo-heptameric structure (Figure 7). During the conformational change the NACHT domains which are responsible for the oligomerization become exposed due to the domain rearrangement and the uCARD effector domains become accessible for the interacting partner molecules.

## Conclusions

Although structures of several LRR-containing proteins have been solved so far [10] the lack of crystal structures of NLRs initiated several individual projects to model one member of the family. There have been some approaches that aimed to generate models for all members of the family [4,8,12].

In our study presented here, homology modeling of the full-length protein was completed by the sequence alignment of NLRC5 and some NLRC5-related proteins (NLRC1, NLRC2, NLRC3, RI and C2TA). We analyzed the consensus patterns in the sequences of LRRs and recognized two typical consensus sequences in the LRRs of NLRC5. However, NLRC5 has unusual N- and C-terminal domains, therefore, it has not been modeled in previous systematic studies and no homology model was available for full-length human NLRC5 until now. In the present study we provide a model for the full-length human NLRC5 in its closed conformation, furthermore, using opened conformational monomers, a homo-heptameric model was also built up.

NLRC5 is an intensively studied receptor with various functions, including the capability of transcriptional regulation of MHC class I, and its regulatory role in various signal transduction pathways has also been reported. NLRC5 has been reported to inhibit NFкB and type I interferon signaling by binding to IKKα/β and retinoic acid-inducible gene I (RIG-I)-like receptors, respectively. It has been also shown that the LRR region of NLRC5 (Ala900-Arg1329) is responsible for mediating the interaction [33]. By modeling of NLRC5 structure, we may contribute to the better understanding of potential interactions of NLRC5 with other proteins, furthermore we may provide molecular tools for future drug designs.

Our model can aid in better interpretation of NLRC5 structure studies in the future, identify structurally or functionally critical residues of the molecule and may assist in design of truncated forms of NLRC5 for further understanding its functions.

## Competing interests

The authors declare that they have no competing interests.

## Authors’ contributions

This study was conceived and managed by JT. Homology modeling was performed by PB, sequence alignments were performed by PB and JM. JM, SB and JT participated in scientific discussions and evaluation of results. JM generated the figures and JM, SB and JT drafted the manuscript. JM, SB and JT read and approved the final manuscript.

## Authors’ information

This paper is dedicated to the memory of Dr. Péter Bagossi. Péter passed away in July of 2011, and the results presented here were among his last ones. The coauthors of this paper regard this manuscript as a memoriam of a very special colleague.

## Supplementary Material

Additional file 1 **Result of secondary structure prediction performed by using PredictProtein server.** Predicted secondary structural organization is indicated below the sequence of full-length NLRC5. α-helices are indicated by "H" and β-sheets are indicated by "E". Predicted LRRs are numbered and indicated by arrows below the sequence. Click here for file

Additional file 2 **Schematic representation of human NLRC5 and the template structures used for homology modeling.** Upper part: Schematic representation of domain architecture of human NLRC5 protein. Lower part: Schematic representation of template structure parts used for homology modeling of the opened and closed conformational NLRC5 protein. Regions of Apaf-1 (1Z6T.pdb and 3IYT.pdb) and ribonuclease inhibitor (1Z7T.pdb) proteins used for homology modeling are indicated, together with the values of sequence identities between the target and template sequences (%). Click here for file

Additional file 3 **Sequences aligned for the homology modeling of full-length human NLRC5 protein.** Sequence of full-length NLRC5 protein is numbered (black). The homology modeling of N-terminal domains of the closed conformational human NLRC5 protein was performed based on the crystal structure of Apaf-1 protein [1Z6T.pdb] (purple). With the exception of the uCARD domain, the N-terminal domains of the opened conformational human NLRC5 were modeled using the structure of the apoptosome-procaspase-9 CARD complex as template [3IYT.pdb] (orange). The uCARD domain of opened conformational NLRC5 was built up using the predicted structure of uCARD domain of closed conformational NLRC5. The LRR domain of human NLRC5 was predicted based on the X-ray structure of human ribonuclease inhibitor [1Z7X.pdb] (blue, red and green). Some α-helices of Apaf-1 protein were completed before homology modeling using the Biopolymer module of Sybyl (brown). A possible conformation of the SH-LRR linker region was optimized after homology modeling by a molecular dynamics procedure using Sybyl (underlined). Identical (*) and similar residues (":" and ".") are indicated, using the similarity defaults of CLUSTAL X program. Click here for file
