# Supplementary material for: A molecular model of the full-length human NOD-like receptor family CARD domain containing 5 (NLRC5) protein
Source: BMC Bioinformatics. 2013 Sep 17;14:275. doi: 10.1186/1471-2105-14-275 (PMC3848420; doi:10.1186/1471-2105-14-275)
Supplement: Additional file 1 — Result of secondary structure prediction performed by using PredictProtein server. Predicted secondary structural organization is indicated below the sequence of full-length NLRC5. α-helices are indicated by "H" and β-sheets are indicated by "E". Predicted LRRs are numbered and indicated by arrows below the sequence. [file 1471-2105-14-275-S1.pdf]

```
1...,....10..,....20..,....30..,....40..,....50..,....60..,....70..,....80
MDPVGLQLGNKNLWSCLVRLLTKDPEWLNAKMKFFLPNTDLDSRNETLDPEQRVILQLNKLHVQGSDTWQSFIHCVCMQL
            HHHHHHHHH    HHH    EE                    EEEHHHHH      HHHHHHHHHHHH

....,....90..,....100.,....110.,....120.,....130.,....140.,....150.,....160
EVPLDLEVLLLSTFGYDDGFTSQLGAEGKSQPESQLHHGLKRPHQSCGSSPRRKQCKKQQLELAKKYLQLLRTSAQQRYR
     HHHHHHHHH     HHHHHHHHHH      HHHHH                    HHHHHHHHHHHHHHHHHHHH

....,....170.,....180.,....190.,....200.,....210.,....220.,....230.,....240
SQIPGSGQPHAFHQVYVPPILRRATASLDTPEGAIMGDVKVEDGADVSISDLFNTRVNKGPRVTVLLGKAGMGKTTLAHR
HH           HHHHHHHHHH                                  HHHH          EEEEE      HHHHHHH

....,....250.,....260.,....270.,....280.,....290.,....300.,....310.,....320
LCQKWAEGHLNCFQALFLFEFRQLNLITRFLTPSELLFDLYLSPESDHDTVFQYLEKNADQVLLIFDGLDEALQPMGPDG
HHHHHHH       EEEEEEEHHHHH      HHHHHHHH       HHHHHHHHHH    EEEEEE

....,....330.,....340.,....350.,....360.,....370.,....380.,....390.,....400
PGPVLTLFSHLCNGTLLPGCRVMATSRPGKLPACLPAEAAMVHMLGFDGPRVEEYVNHFFSAQPSREGALVELQTNGRLR
   HHHHHHHHHH       EEEEE    HHHHH      EEEEEE  HHHHHHHHHHH    HHHHHHHHHHHH   HH

....,....410.,....420.,....430.,....440.,....450.,....460.,....470.,....480
SLCAVPALCQVACLCLHHLLPDHAPGQSVALLPNMTQLYMQMVLALSPPGHLPTSSLLDLGEVALRGLETGKVIFYAKDI
HHHH  HHHHHHHHHHHHHH       HHHHHHHHHHHHHHH       HHHHHHHHHHHHHHHH   EEE  HHH

....,....490.,....500.,....510.,....520.,....530.,....540.,....550.,....560
APPLIAFGATHSLLTSFCVCTGPGHQQTGYAFTHLSLQEFLAALHLMASPKVNKDTLTQYVTLHSRWVQRTKARLGLSDH
H      HHHHHHHHHHHH           EEEHHHHHHHHHHHHHHHHH        HHHHHHHHHHHHHHHHHH   HHH

....,....570.,....580.,....590.,....600.,....610.,....620.,....630.,....640
LPTFLAGLASCTCRPFLSHLAQGNEDCVGAKQAAVVQVLKKLATRKLTGPKVVELCHCVDETQEPELASLTAQSLPYQLP
HHHHHHH   HHHHHHHHHHH          HHHHHHHHHH        HHHHHHHHHHHH   HHHHHHHHHH     H

....,....650.,....660.,....670.,....680.,....690.,....700.,....710.,....720
FHNFPLTCTDLATLTNILEHREAPIHLDFDGCPLEPHCPEALVGCGQIENLSFKSRKCGDAFAEALSRSLPTMGRLQMLG
HHH     HHHHHHHHHHHH                HHHHHH    1          HHHHHHHHHH          2EEE

....,....730.,....740.,....750.,....760.,....770.,....780.,....790.,....800
LAGSKITARGISHLVKALPLCPQLKEVSFRDNQLSDQVVLNIVEVLPHLPRLRKLDLSSNSICVSTLLCLARVAVTCPTV
       HHHHHHHHHHHHH    3 EEE        HHHHHHHHHHHH     EEEE  4     HHHHHHHHHHH     5

....,....810.,....820.,....830.,....840.,....850.,....860.,....870.,....880
RMLQAREADLIFLLSPPTETTAELQRAPDLQESDGQRKGAQSRSLTLRLQKCQLQVHDAEALIALLQEGPHLEEVDLSGN
EEEE     HHHHH  HHHHHHH    6                 HHHEEEE   7   HHHHHHHHHHHHH       EEE  8

....,....890.,....900.,....910.,....920.,....930.,....940.,....950.,....960
QLEDEGCRLMAEAASQLHIARKLDLSDNGLSVAGVHCVLRAVSACWTLAELHISLQHKTVIFMFAQEPEEQKGPQERAAF
   HHHHHHHHHHHH     EEEE  9     HHHHHHHHHHHHH    EEEE  10   EEEE          11 HH

....,....970.,....980.,....990.,....1000.,....1010.,....1020.,....1030.,....1040
LDSLMLQMPSELPLSSRRMRLTHCGLQEKHLEQLCKALGGSCHLGHLHLDFSGNALGDEGAARLAQLLPGLGALQSLNLS
HHHHH           EEEE   12  HHHHHHHHHHHHHHHHHHHH   13     HHHHHHHHHHHH       EE 14

....,....1050.,....1060.,....1070.,....1080.,....1090.,....1100.,....1110.,....1120
ENGLSLDAVLGLVRCFSTLQWLFRLDISFESQHILLRGDKTSRDMWATGSLPDFPAAAKFLGFRQRCIPRSLCLSECPLE
     HHHHHHHHHHHH       HHHH  15           HHHHHHHHH   16      HHHHH    EEE  17

....,....1130.,....1140.,....1150.,....1160.,....1170.,....1180.,....1190.,....1200
PPSLTRLCATLKDCPGPLELQLSCEFLSDQSLETLLDCLPQLPQLSLLQLSQTGLSPKSPFLLANTLSLCPRVKKVDLRS
  HHHHHHHHHHH     EEE 18        HHHHHHHH      EEEE  19     HHHHHHHHHH    20
```

```
....,....1210.,....1220.,....1230.,....1240.,....1250.,....1260.,....1270.,....1280
LHHATLHFRSNEEEEGVCCGRFTGCSLSQEHVESLCWLLSKCKDLSQVDLSANLLGDSGLRCLLECLPQVPISGLLDLSH
                HHHHHHH  21        HHHHHHHHHHHH     EEE 22       HHHHHHHHH      23 EEE

....,....1290.,....1300.,....1310.,....1320.,....1330.,....1340.,....1350.,....1360
NSISQESALYLLETLPSCPRVREASVNLGSEQSFRIHFSREDQAGKTLRLSECSFRPEHVSRLATGLSKSLQLTELTLTQ
      HHHHHHHHHHHHHHHHH    24        HHH         EEE      25 HHHHHHHHHHH      EEEE  26

....,....1370.,....1380.,....1390.,....1400.,....1410.,....1420.,....1430.,....1440
CCLGQKQLAILLSLVGRPAGLFSLRVQEPWADRARVLSLLEVCAQASGSVTEISISETQQQLCVQLEFPRQEENPEAVAL
      HHHHHHHHHH       EEE  27     HHHHHHHHHHH       EEE  28                   EEEE 29

....,....1450.,....1460.,....1470.,....1480.,....1490.,....1500.,....1510.,....1520
RLAHCDLGAHHSLLVGQLMETCARLQQLSLSQVNLCEDDDASSLLLQSLLLSLSELKTFRLTSSCVSTEGLAHLASGLGH
EE       HHHHHHHHHHHH         EEE  30         HHHHHHHHHHH        EEE   31  HHHHHHHHHHHHH

....,....1530.,....1540.,....1550.,....1560.,....1570.,....1580.,....1590.,....1600
CHHLEELDLSNNQFDEEGTKALMRALEGKWMLKRLDLSHLLLNSSTLALLTHRLSQMTCLQSLRLNRNSIGDVGCCHLSE
     EEE  32    HHHHHHHHHHHHH        EEE   33  HHHHHHHHHHHH          EEE  34      HHHHHHHHH

....,....1610.,....1620.,....1630.,....1640.,....1650.,....1660.,....1670.,....1680
ALRAATSLEELDLSHNQIGDAGVQHLATILPGLPELRKIDLSGNSISSAGGVQLAESLVLCRRLEELMLGCNALGDPTAL
HHHH     EEE  35    HHHHHHHHHHHH      EEEE    36   HHHHHHHHHHHH           EEE 37      HHHH

....,....1690.,....1700.,....1710.,....1720.,....1730.,....1740.,....1750.,....1760
GLAQELPQHLRVLHLPFSHLGPGGALSLAQALDGSPHLEEISLAENNLAGGVLRFCMELPLLRQIDLVSCKIDNQTAKLL
HHHHHHHHHHHHH   38         HHHHHHHHHHHH        EEE   39  HHHHHHHHH         EEEE  40   HHHHHHH

....,....1770.,....1780.,....1790.,....1800.,....1810.,....1820.,....1830.,....1840
TSSFTSCPALEVILLSWNLLGDEAAAELAQVLPKMGRLKRVDLEKNQITALGAWLLAEGLAQGSSIQVIRLWNNPIPCDM
HHHHHH     EEEE 41         HHHHHHHHHHHH        EEE   42  HHHHHHHHHHHH             EEEE  43    H

....,....1850.,....1860.,1866
AQHLKSQEPRLDFAFFDNQPQAPWGT
HHHH         EE
```
