# Supplementary figures and images for: A molecular model of the full-length human NOD-like receptor family CARD domain containing 5 (NLRC5) protein
Source: BMC Bioinformatics. 2013 Sep 17;14:275. doi: 10.1186/1471-2105-14-275 (PMC3848420; doi:10.1186/1471-2105-14-275)

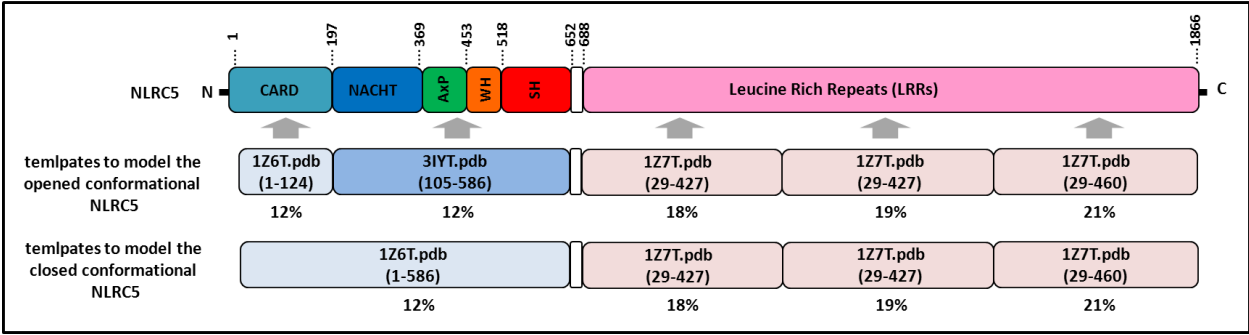

Supplement: Additional file 2 — Schematic representation of human NLRC5 and the template structures used for homology modeling. Upper part: Schematic representation of domain architecture of human NLRC5 protein. Lower part: Schematic representation of template structure parts used for homology modeling of the opened and closed conformational NLRC5 protein. Regions of Apaf-1 (1Z6T.pdb and 3IYT.pdb) and ribonuclease inhibitor (1Z7T.pdb) proteins used for homology modeling are indicated, together with the values of sequence identities between the target and template sequences (%). [file 1471-2105-14-275-S2.pdf]
