# Supplementary material for: A molecular model of the full-length human NOD-like receptor family CARD domain containing 5 (NLRC5) protein
Source: BMC Bioinformatics. 2013 Sep 17;14:275. doi: 10.1186/1471-2105-14-275 (PMC3848420; doi:10.1186/1471-2105-14-275)
Supplement: Additional file 3 — Sequences aligned for the homology modeling of full-length human NLRC5 protein. Sequence of full-length NLRC5 protein is numbered (black). The homology modeling of N-terminal domains of the closed conformational human NLRC5 protein was performed based on the crystal structure of Apaf-1 protein [1Z6T.pdb] (purple). With the exception of the uCARD domain, the N-terminal domains of the opened conformational human NLRC5 were modeled using the structure of the apoptosome-procaspase-9 CARD complex as template [3IYT.pdb] (orange). The uCARD domain of opened conformational NLRC5 was built up using the predicted structure of uCARD domain of closed conformational NLRC5. The LRR domain of human NLRC5 was predicted based on the X-ray structure of human ribonuclease inhibitor [1Z7X.pdb] (blue, red and green). Some α-helices of Apaf-1 protein were completed before homology modeling using the Biopolymer module of Sybyl (brown). A possible conformation of the SH-LRR linker region was optimized after homology modeling by a molecular dynamics procedure using Sybyl (underlined). Identical (*) and similar residues (":" and ".") are indicated, using the similarity defaults of CLUSTAL X program. [file 1471-2105-14-275-S3.pdf]

```
1.......10.......20.......30.......40.......50.......60
MDPVGLQLGNKNLWSCLVRLLTKDPEWLNAKMKFFLPNTDLDSRNETLDPEQRVILQLNK
-----------MDAKARNCLLQHREALEKDIKTSYIMDHMISDGFLTISEEEKVRNEPT-
---VGLQLGNKNLWKARNCLLQHR----------------------TISEEEKVRNEPTK
              ..   ** :     :   . :: :  :..   *:. *::*  : .

61......70.......80.......90...  .....100......110.....118
LHVQGSDTWQSFIHCVCMQLEVPLDLEVLLLSTF--GYDDGFTSQLGAEGKSQPESQLHH
-------QQQRAAMLIKMILKKDNDSYVSFYNALLHEGYKDLAALLHDGI----------
LHVQG----------------------------------KDLAALLHDEGKSQPESQL--
         *     : * *:   *  * : .::     ..::: *

119.              .......130......140......150......160..165
GLKR-------------PHQSCGSSPRRKQCKKQQLELAKKYLQLLRTSAQQRYRSQIPG
----PVVSSSSGKDSVS-----------------------GITSYVRTVLCEGGVPQRPV
------------------------------CKKQQLELAKKYLQYVRTVLCEG-------
----------------------------------------GITSYVRTVLCEGGVPQRPV
                                           . :**   :   .* *

166.170......180  .......190......200......210.........223
SGQPHAFHQVYVPPILR--RATASLDTPEGAIMGDVKVEDGADVSISDLFNTRVNKGPRV
----------------------------------VFVTRKKLVNAIQQKLSKLKGEP-GW
----------------------------------VFVTRKKLVNAIQQKLSKLKGEP-GW
                                     ...    :*.: :..  .:

224...230......240......250   ......260......270......280
TVLLGKAGMGKTTLAHRLCQKWAEGHLNC---FQALFLFEFRQLNLITRFLTPSELLFDL
VTIHGMAGCGKSVLAAEAVRDHSLLEGCFPGGVHWVSVGKQDKSGLLMKLQNLCTRLDQD
VTIHGMAGCGKSVLAAEAVRDHSLLEGCFPGGVHWVSVGKQDKSGLLMKLQNLCTRLDQD
..: * ** **:.** .  :. :  .      .: : : :  : .*: :: . .  * :

281.         .....290......300......310.....       ..320..325
YLSP---------ESDHDTVFQYLEKNADQVLLIFDGLDEALQPMG------PDGPGPVL
ES--FSQRLPLNIEEAKDRLRILMLRKHPRSLLILDDVWDSWVLKA------FDSQCQ--
ES--FSQRLPLNIEEAKDRLRILMLRKHPRSLLILDDVWDSWVLKA------FDSQCQ--
             *. :* :   : ::  : ***:*.: ::    .       *.

326.330.  ......340......350.         ......360......370..375
TLFSHLCN--GTLLPGCRVMATSRPGKLPA--------CLPAEAAMVHMLGFDGPRVEEY
------------------ILLTTRDKSVTD----------SVMGPKYVVPVESSLGKEKG
------------------ILLTTRDKSVTD----------SVMGPKYVVPVESSLGKEKG
                  :: *:*  .:.           .. ..   :   ..   *:
```

```
376.380......        .390.......400.  ......410....  ...420.424
VNHFFSAQPSREG-------ALVELQTNGRLRSLC--AVPALCQVACLCL--HHLLPDHA
LEILSLFVNMKK--------ADLPEQAHSIIKECK--GSPLVVSLIGALLRDFPNRWEYY
LEILSLFVNMKK--------ADLPEQAHSIIKECK--GSPLVVSLIGALLRDFPNRWEYY
:: :      ::         * :  *::. :      . * : .:    *  .    ::

425..430.......440.......450.              ......460......469
PGQSVALLPNMTQLYMQMVLALSPPGHLP--------------TSSLLDLGEVALRGLE
LKQLQNKQFKRIRKSSSYDYEALDEAMSISVEMLREDIKDYYTDLSILQKDVKVPTKVLC
LKQLQNKQFKRIRKSSSYDYEALDEAMSISVEMLREDIKDYYTDLSILQKDVKVPTKVLC
  *             .                            * * .  :*. : *

470.......480.......490.......500...    ....510.......520..525
TGKVIFYAKDIAPPLIAFGATHSLLTSFCVCTGPGH----QQTGYAFTHLSLQEFLAALH
ILWDM-ETEEVEDILQEF-VNKSLL--FCDRNGKSFRYYLHDLQVDFLTEKNCSQLQDLH
ILWDM-ETEEVEDILQEF-VNKSLL--FCDRNGKSFRYYLHDLQVDFLTEKNCSQLQDLH
    :  ::::   *  *  ..:***  **   .* ..    ::    *   .  . *  **

526.530.......540..       .....550.......560.......570.....578
LMASPKVNKDTLTQYVTLH-------SRWVQRTKARLGLSDHLPTFLAGLASCTCRPFLS
KKIITQFQRYHQPHTLSPDQEDCMYWYNFLAYHMASAKMHKELCALMFSLDWIKAKTELV
KKIITQFQRYHQPHTLSPDQEDCMYWYNFLAYHMASAKMHKELCALMFSLDWIKAKTELV
    .:.::   .: :: .         .::    *   :  ..* ::: .*    ..:. *

579.......590.......600.......610.......620.......630.....638
HLAQGNEDCVGAKQAAVVQVLKKLATRKLTGPKVVELCHCVDETQEPELASLTAQSLPYQ
GPAHLIHEFVEYR---HILDEKDCAVSENFQEFLSLNGHLLGRQPFPNIVQLGLCEPETS
GPAHLIHEFVEYR---HILDEKDCAVSENFQEFLSLNGHLLGRQPFPNIVQLGLCEPETS
  *:  .: *  :    :   *. *. :     :    * :..   *:...*    .   .

639.......650.......660.......670.......680....687
LPFHNFPLTCTDLATLTNILEHREAPIHLDFDGCPLEPHCPEALVGCGQ
EVYQQAKLQAKQ-------------------------------------
EVYQQAKLQAKQ-------------------------------------
  :::  * ..:

688.........700.......710..715
IENLSFKSRKCGDAFAEALSRSLPTMGR
CQVVRLDDCGLTEARCKDISSALRVNPA
 : : :..    :* .: :* :* .

716.720.......730....... 740
LQMLGLAGSKITARGISHLVKALP-LCPQ
LAELNLRSNELGDVGVHCVLQGLQTPSCK
*  *.* ..::   *:  :::.*   . :
```

```
744...750.......760.......770
LKEVSFRDNQLSDQVVLNIVEVLPHLPR
IQKLSLQNCCLTGAGCGVLSSTLRTLPT
::::*:::  *:.     : ..*  **

772.....780.......790... ...799
LRKLDLSSNSICVSTLLCLARVAV-TCPT
LQELHLSDNLLGDAGLQLLCEGLLDPQCR
*::*.**.* :  : *  *..  : .

800......       .810.......820
VRMLQAREA-------DLIFLLSPPTET
LEKLQLEYCSLSAASCEPLASVLRAKPD
:. ** . .       : :  :  ..

821......830.......840    ..844
TAELQRAPDLQESDGQRKGAQ-----SRS
FKELTVSNNDINEAGVRVLCQGLKDSPCQ
  **  : :  :. * *  .*     . .

845...850.......860........871
L-TLRLQKCQLQVHDAEALIALLQEGPH
LEALKLESCGVTSDNCRDLCGIVASKAS
* :*:*:.* :  .:.. * .:: . .

872.....880.......890.. ....899
LEEVDLSGNQLEDEGCRLMAEAA-SQLHI
LRELALGSNKLGDVGMAELCPGLLHPSSR
*.*: *..*:* * *   :. .

900.......910.......920....927
ARKLDLSDNGLSVAGVHCVLRAVSACWT
LRTLWIWECGITAKGCGDLCRVLRAKES
 *.* : : *::. *   : *.: *  :

928....   .....940..........953
LAELHIS---LQHKTVIFMFAQEPEEQKG
LKELSLAGNELGDEGARLLCETLLEPGCQ
* ** ::   * .: . ::     *

954...    960.......970  ..975
PQERAA----FLDSLMLQMPSEL--PLS
LESLWVKSCSFTAACCSHFSSVLAQNRF
 :.  .    *  :   ::.* *
```

```
976.980.......990.......1000..1006
SRRMRLTHCGLQEKHLEQLCKALGGSCHLGH
LLELQISNNRLEDAGVRELCQGLGQPGSV--
  .:::::  *::  :.:**:.** .  :

1007..........1020......1030
L-HLDFSGNALGDEGAARLAQLLPGLGA
LRVLWLADCDVSDSSCSSLAATLLANHS
*  * ::.  :.*...: **  * .  :

1034..1040......1050......1060
LQSLNLSENGLSLDAVLGLVRCFSTLQW-
LRELDLSNNCLGDAGILQLVESVRQPGCL
*:.*:**:* *.  .:* **...

1062...       .1070........1082
LFRLDIS-------FESQHILLRGDKTS
CQVVRLDDCGLTEARCKDISSALRVNPA
   : :.         .:       :.:

 1083....1090......1100. ..1107
-RDM-WATGSLPDFPAAAKFLGFR-QRC-
LAELNLRSNELGDVGVHCVLQGLQTPSCK
  ::   :..* *. . . : *::   *

1108........1120......1130...1137
IPRSLCLSECPLEPPSLTRLCATLKDCPGP
I-QKLSLQNCCLTGAGCGVLSSTLRTLPT-
* :.*.*.:* *  ..   *.:**:  *

1138.........1150......1160.
L-ELQLSCEFLSDQSLETLLDCLPQLPQ-
LQELHLSDNLLGDAGLQLLCEGLLDPQCR
* **:** ::*.* .*: * : *

1165.1170......1180......1190
LSLLQLSQTGLSPKSPFLLANTLSLCPR
LEKLQLEYCSLSAASCEPLASVLRAKPD
*. ***.  .**. *   **..*   *

1193...  1200......1210 ....1216
VKKVDLR--SLHHATLHFRSN---EEEEG
FKELTVSNNDINEAGVRVLCQGLKDSPCQ
.*:: :   .::.* ::. .:    :.
```

```
1217.........1230......1240
VCCGRFTGCSLSQEHVESLCWLLSKCKD
LEALKLESCGVTSDNCRDLCGIVASKAS
: . :: .*.::.:: ..** :::.  .

1245.1250......1260.......1271
LSQVDLSANLLGDSGLRCLLECLPQVP--
LRELALGSNKLGDVGMAELCPGLLHPSSR
* :: *.:* *** *:  *   * : .

1272....1280......1290......1300
ISGLLDLSHNSISQESALYLLETLPSCPR
LRTLWIWECGITAKGCGDLCRVLRAKES-
:  *   . .  :: ..       .. .

1301...    ..1310   ......1320
VREASVN---LGSEQS---FRIHFSREDQ
LKELSLAGNELGDEGARLLCETLLEPGCQ
::* *:    **.* :    .  :.   *

1324..1330......1340......1350
AGKTLRLSECSFRPEHVSRLATGLSKSLQ
L-ESLWVKSCSFTAACCSHFSSVLAQNRF
  ::* :..*** .   *:::: *::.

1353...1360......1370......1380
LTELTLTQCCLGQKQLAILLSLVGRPAG-
LLELQISNNRLEDAGVRELCQGLGQPGSV
* ** :::  * :  :  * . :*:*..

1381.....1390......1400.....1409
LFSLRVQEPWADRARVLSLLEVCAQASGS
LRVLWLADCDVSDSSCSSLAATLLANHS-
*  * : :  .. :   **  .     .

1410......1420......1430..1436
VTEISISETQQQLCVQLEFPRQEENPE--
LRELDLSNNCLGDAGILQLVESVRQPGCL
: *:.:*:.    .  *:: .. .:*

1437.........1450......1460.
AVALRLAHCDLGAHHSLLVGQLMETCAR
CQVVRLDDCGLTEARCKDISSALRVNPA
. .:** .*.*   :.  :.. :.. .
```

```
1465.1470......1480......1490.1495
LQQLSLSQVNLCEDDDASSLLLQSLLLSLSE-
LAELNLRSNELG---DVGVHCVLQGLQTPSCK
* :*.* . :*    * .   :   *   *

1496.........1510......1520
LKTFRLTSSCVSTEGLAHLASGLGHCHH
IQKLSLQNCCLTGAGCGVLSSTLRTLPT
::.: * ..*::  * . *:* *

1524..1530......1540......1550
LEELDLSNNQFDEEGTKALMRALEGKWM-
LQELHLSDNLLGDAGLQLLCEGLLDPQCR
*:**.**:* :.: * : * ..* .

1552....1560......1570.....1579
LKRLDLSHLLLNSSTLALLTHRLSQMTC
LEKLQLEYCSLSAASCEPLASVLRAKPD
*::*:*.:  *.:::    *:  *   .

1580......1590......1600...1607
LQSLRLNRNSIGDVGCCHLSEALRAATS-
FKELTVSNNDINEAGVRVLCQGLKDSPCQ
::.* :..*.*.:.*   *.:.*: :..

1608........1620......1630.1635
LEELDLSHNQIGDAGVQHLATILPGLPE
LEALKLESCGVTSDNCRDLCGIVASKAS
** *.*.   : . . :.*. *:.. ..

1636.........1650......1660
LRKIDLSGNSISSAGGVQLAESLVLCRR-
LRELALGSNKLGDVGMAELCPGLLHPSSR
**:: *..*.:...* .:*. .*:

1664..1670......1680.....1689
LEELMLGCNALGDPTALGLAQELPQH--
LRTLWIWECGITAKGCGDLCRVLRAKES
*. * :   .:    . .*.: *  :

1690......1700......1710...1717
LRVLHLPFSHLGPGGALSLAQALDGSPH-
LKELSLAGNELGDEGARLLCETLLEPGCQ
*: * *. ..**  **  *.::*  .
```

```
1718........1730.......1740
LEEISLAENNLAG-GVLRFCMELPL---
LESLWVKSCSFTAACCSHFSSVLAQNRF
**.: : . .::.    :*.  *.

1742....1750......1760.....1769
LRQIDLVSCKIDNQTAKLLTSSFTSCPA-
LLELQISNNRLEDAGVRELCQGLGQPGSV
* :::: . ::::  .: * ..: .  :

1770......1780......1790...1797
LEVILLSWNLLGDEAAAELAQVLPKMGR
LRVLWLADCDVSDSSCSSLAATLLANHS
*.*: *:   :.*.:.:.** .*

1798........1810......1820.1825
LKRVDLEKNQITALGAWLLAEGLAQGSS-
LRELDLSNNCLGDAGILQLVESVRQPGCL
*:.:**.:* :   *   *.*.: * ..

1826..........1840......1850......1860..1866
IQVIRLWNNPIPCDMAQHLKSQEPRLDFAFFDNQPQAPWGT
LEQLVLYDIYWSEEMEDRLQALEKDKPSLRVIS--------
:: : *::   . :* ::*:: *       . .
```
